# The Cyanobacterial Hepatotoxin Microcystin Binds to Proteins and Increases the Fitness of *Microcystis* under Oxidative Stress Conditions

**DOI:** 10.1371/journal.pone.0017615

**Published:** 2011-03-18

**Authors:** Yvonne Zilliges, Jan-Christoph Kehr, Sven Meissner, Keishi Ishida, Stefan Mikkat, Martin Hagemann, Aaron Kaplan, Thomas Börner, Elke Dittmann

**Affiliations:** 1 Institute of Biology, Department of Molecular Ecology, Humboldt University, Berlin, Germany; 2 Institute of Biochemistry and Biology, Department of Microbiology Potsdam-Golm, University of Potsdam, Potsdam, Germany; 3 Leibniz Institute for Natural Product Research and Infection Biology, Hans Knöll Institute, Jena, Germany; 4 Medical Faculty, Core Facility Proteome Analysis, University of Rostock, Rostock, Germany; 5 Institute of Biology, Department of Plant Physiology, University of Rostock, Rostock, Germany; 6 Department of Plant and Environmental Sciences, The Hebrew University of Jerusalem, Jerusalem, Israel; 7 Humboldt University, Institute of Biology, Department of Genetics, Berlin, Germany; National Institutes of Health, United States of America

## Abstract

Microcystins are cyanobacterial toxins that represent a serious threat to drinking water and recreational lakes worldwide. Here, we show that microcystin fulfils an important function within cells of its natural producer *Microcystis*. The microcystin deficient mutant Δ*mcyB* showed significant changes in the accumulation of proteins, including several enzymes of the Calvin cycle, phycobiliproteins and two NADPH-dependent reductases. We have discovered that microcystin binds to a number of these proteins *in vivo* and that the binding is strongly enhanced under high light and oxidative stress conditions. The nature of this binding was studied using extracts of a microcystin-deficient mutant *in vitro*. The data obtained provided clear evidence for a covalent interaction of the toxin with cysteine residues of proteins. A detailed investigation of one of the binding partners, the large subunit of RubisCO showed a lower susceptibility to proteases in the presence of microcystin in the wild type. Finally, the mutant defective in microcystin production exhibited a clearly increased sensitivity under high light conditions and after hydrogen peroxide treatment. Taken together, our data suggest a protein-modulating role for microcystin within the producing cell, which represents a new addition to the catalogue of functions that have been discussed for microbial secondary metabolites.

## Introduction

*Microcystis* is a unicellular colonial cyanobacterium frequently developing blooms in freshwater habitats [1]. Buoyant colonies may form thick scums at the surface of lakes where they are exposed to high light. Even under these high irradiances, *Microcystis* shows high photosynthesis rates, thereby maintaining numerical dominance in eutrophic waters when physico-chemical conditions favour bloom formation [2].

*Microcystis* is known for its production of the potent hepatotoxin microcystin, a nonribosomal peptide that inhibits eukaryotic protein phosphatases of types 1 and 2A. The cyclic heptapeptide is capable of forming a covalent bond to a cysteine moiety of the catalytic domain of these enzymes that are key components of signalling pathways [3]. Microcystin intoxications can affect humans and livestock as well as various cyanobacterial grazers such as *Daphnia* (see [4] and references therein). Nevertheless, the role of microcystins as a feeding deterrent is questionable since significant amounts of microcystin are released from the cells only after lysis. Moreover, feeding experiments revealed that *Microcystis* strains can inhibit grazing activity of *Daphnia* regardless of the occurrence of microcystins [5], [6]. Finally, a phylogenetic analysis of the microcystin biosynthesis genes (*mcy*) among diverse cyanobacterial genera revealed that they co-evolved with house-keeping genes and clearly preceded the appearance of the metazoans, i.e. well ahead of the potential grazers [7].

Progress in the clarification of the biological role of microcystin was further fuelled by studies which examined the response of *Microcystis* itself to externally added microcystin. These experiments provided us with evidence that microcystins act as infochemicals. Their release to the medium by lysing cells signals to the rest of the population that they are facing stress conditions [8]. An extracellular role of microcystin may also account for observed differences in the accumulation of cell surface proteins and colony formation between *Microcystis* PCC 7806 wild type and its microcystin-free Δ*mcyB* mutant [9], [10].

Few other studies pointed to an intracellular function of microcystins. The Δ*mcyB* mutant showed clear differences in pigmentation [11] and was proposed to show altered adaptation to low inorganic carbon concentrations [12]. Further hints were obtained from the analysis of the transcriptional regulation of the *mcy* cluster. Strong illumination or iron limitation led to increased accumulation of *mcy* mRNA [13], [14]. As expected, the rise in the transcription of the *mcy* genes under high light conditions was accompanied by a corresponding increase in the amount of McyB [15]. However, an increase of microcystin itself could not be observed in these studies; we often observed a decline in its level under conditions where an enhanced transcription of the *mcy* genes was detected ([13] and unpublished data).

Here, we provide evidence that the apparent loss of microcystin is likely the consequence of a specific and covalent binding of the toxin to various proteins. This binding is strongly enhanced under high light conditions. Absence of microcystin leads to differences in the accumulation of a number of these proteins in *Microcystis.* Our data suggest an important intracellular function of microcystin during acclimation of *Microcystis* to high light and oxidative stress conditions.

## Results

### Loss of microcystin leads to an altered accumulation of specific proteins in *M. aeruginosa* PCC 7806

**Figure 1:**
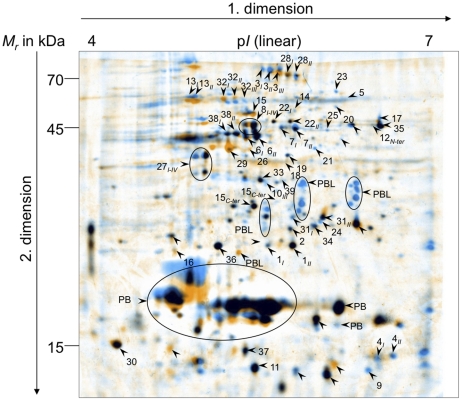
Proteomic comparison of *Microcystis aeruginosa* PCC 7806 wild type and microcystin-deficient Δ*mcyB* mutant. Proteome of soluble fraction of *Microcystis aeruginosa* PCC 7806 wild type (WT, blue colouring) and microcystin-deficient Δ*mcyB* mutant (orange colouring) after exposition to high light of 70 µmol photons m^−2^s^−1^ for 2 hours. Gels were obtained after separation of soluble protein extracts (400 µg) by 2-DE (first dimension: IEF pH range 4–7 linear, second dimension: SDS-PAGE applying 12.5% acrylamide) and Coomassie-staining. Representative well resolved gels of WT and Δ*mcyB* were grouped, respectively, warped and fused to one gel via Delta2D version 4.0 (DECODON, Greifswald, Germany). Differential protein spots and selected non-differential protein spots analyzed for standardization are indicated with arrows. Highly abundant phycobiliproteins (PB), associated linker proteins (PBL) as well as proteins of more than three isoforms are encircled. Protein identities are given in Table 1 and Table S1.

In an earlier study we showed a fast rise in the accumulation of *mcy* mRNA encoding microcystin biosynthesis enzymes in *Microcystis* cells exposed to light intensities higher than about 50 µmol photons m^−2^ s^−1^ [13]. We now performed a proteomic comparison of protein extracts from wild type and Δ*mcyB* mutant, unable to produce microcystins, to examine possible consequences of the loss of microcystin formation in the mutant. Cultures grown at 16 µmol photons m^−2^ s^−1^ (control light) to mid logarithmic phase were exposed for 2 h to 70 µmol photons m^−2^ s^−1^ (high light) or darkness. Several differences in the cytosolic proteins composition were observed under both high light and darkness (Table 1, Fig. 1 and Table S1). From 492 detected protein spots 21 (4.3%) are repressed and 83 (17.0%) are induced in the mutant under light condition (L). Under dark conditions (D) 37 spots (7.5%) were repressed and 22 spots were induced (4.5%) in Δ*mcyB* mutant (Table 1). Similar results were obtained in six replicate experiments (see Materials and Methods). Protein spots showing differences in abundance were excised from gels, trypsinated and analysed by MALDI-TOF mass spectrometry. The signals were then compared with theoretical masses of tryptic peptides as predicted from a protein database of *M. aeruginosa* PCC 7806 (EMBL accession numbers AM778843 to AM778958) and the SWALL database using the taxonomy filter Eubacteria. Using this approach 60% and 90% of the differentially expressed spots could be identified for high light (L) and dark (D) conditions, respectively (Table 1 and S1). The analyses revealed that in many cases WT and Δ*mcyB* mutant accumulate different isoforms of the same proteins (see Figure 1 and Table S1). In addition to the occurrence of different isoforms several proteins showed also differences in their abundance. Notably, the list of proteins showing altered accumulation (Table 1) includes a number of proteins related to photosynthesis; in particular enzymes of the Calvin cycle. Many of the spots could be assigned to phycobiliproteins (PB) or their linkers (PBL). Due to the high amount PB proteins and their partition in various isoforms we omitted them from our quantitative analysis. It was apparent, however, that the wild type strain accumulated a higher number of isoforms of PBL proteins (see Figure 1). The list of proteins with varying abundance also includes known regulatory proteins, such as two variants of CP12 [16]. Other candidates belong to the categories of protein synthesis, cellular processes/detoxification, central intermediate metabolism, biosynthesis of amino acids and co-factors, energy and fatty acid metabolism and hypothetical proteins (Table 1). For selected candidates, differences in protein or protein isoform accumulation could be confirmed in Western blot experiments (Figure S1). The large number of differentially expressed proteins related to primary metabolism was unexpected for a mutant impaired in the production of a secondary metabolite.

**Table 1:** List of proteins accumulated or repressed more than two-fold in the microcystin free Δ*mcy*B mutant in comparison to wild type *M. aeruginosa* PCC 7806 (WT) after short term exposure to high light (L) or dark (D) conditions.

Spot no.^1^	Δ*mcy*B *versus* WT		ORF^2^	Identity/Protein function^3^	Category
	L	D			
1*_I-II_*	+6.2	−3.0	A8YJ50_MICAE	CBS domain containing membrane protein, CP12 like polypeptide	Photosynthesis
2	±0	+2.9	A8YBT0_MICAE	CBS domain containing membrane protein, CP12 like polypeptide	Photosynthesis
3*_I-III_*	+3.6	±0	A8YNI2_MICAE	Transketolase	Photosynthesis
4*_I-II_*	−2.0	−2.0	A8YF93_MICAE	small subunit of RubisCO	Photosynthesis
5	−2.0	−2.0	A8YF91_MICAE	large subunit of RubisCO	Photosynthesis
6*_I-II_*	±0	−2.2	A8YD92_MICAE	Phosphoribulokinase	Photosynthesis
7*_I-II_*	−3.1	−2.6	A8YJ16_MICAE	fructose-bisphosphate aldolase class II	Photosynthesis
8*_I-IV_*	−2.0	+4.4	A8YJZ4_MICAE	fructose-1,6-/sedoheptulose-1,7-bisphosphatase	Photosynthesis
9	−3.7	−5.7	A8YF87_MICAE	carbon dioxide concentrating mechanism protein CcmK	Photosynthesis
PB^4^	n.q.	n.q.	A8YJM8_MICAE	phycocyanin alpha subunit	Photosynthesis
PBL^4^	n.q.	n.q.	A8YJM9_MICAE	phycobilisome 32.1 kDa linker polypeptide, phycocyanin-associated, Rod1	Photosynthesis
12*_N-ter_*	±0	−2.0	A8YD60_MICAE	ATP synthase CF1 alpha chain AtpA	Photosynthesis
13*_I_*	+2.1	−2.0	A8YHP0_MICAE	ATP synthase CF1 beta subunit AtpB	Photosynthesis
13*_II_*	+3.1	−2.0	A8YHP0_MICAE	ATP synthase CF1 beta subunit AtpB	Photosynthesis
14	+7.7	−2.4	A8YJB2_MICAE	thioredoxin reductase	Photosynthesis
15	+2.2	+2.4	A8YIW2_MICAE	translation elongation factor EF-Tu	Protein biosynthesis
15*_C-ter_*	+3.4	+2.4	A8YIW2_MICAE	translation elongation factor EF-Tu	Protein biosynthesis
16	−6.5	±0	A8YLU5_MICAE	Peroxiredoxin	Cellular processes
17	+2.6	+4.3	A8YF09_MICAE	glucose-1-phosphate adenylyltransferase	Central intermediary metabolism
18	±0.	+2.4	A8YBS4_MICAE	cysteine synthase	Amino acid biosynthesis
19	−2.0	−2.0	A8YC37_MICAE	ketol-acid reductoisomerase	Amino acid biosynthesis
20	±0	−3.0	A8YMK3_MICAE	glutathione reductase	Biosynthesis of cofactors, prosthetic groups and carriers
21	±0	−4.5	A8YMU9_MICAE	uroporphyrinogen decarboxylase	
22*_I_*	+4.3	−2.0	A8YEP8_MICAE	adenosylhomocysteinase	Energy metabolism
22*_II_*	+3.5	−2.0	A8YEP8_MICAE	adenosylhomocysteinase	Energy metabolism
23	−2.0	±0	A8YF09_MICAE	glucose-6-phosphate isomerase	Energy metabolism
24	+3.5	−2.0	A8YJ06_MICAE	3-oxoacyl-[acyl carrier protein] reductase; FabG2	Fatty acid, phospholipid and sterol metabolism
25	±0	−2.0	A8YJ05_MICAE	acetoacetyl-CoA thiolase	
26	−2.0	+3.0	A8YFM0_MICAE	probable oxidoreductase	Unknown function
27*_I-IV_*	+3.2	+2.1	A8YG81_MICAE	water-soluble carotenoid protein	Unknown function
28*_I-II_*	−2.0	±0	A8YM76_MICAE	hypothetical protein	Unknown function
29	+35.3	+9.4	A8YDB5_MICAE	hypothetical protein, MrpA	Unknown function
30	+2.0	±0	A8YHX5_MICAE	hypothetical protein, MrpC	Unknown function
31*_I−II_*	±0	+8.3	A8YDG4_MICAE	probable lysozyme	Unknown function
32*_I_*	+2.8	±0	A8YEX3_MICAE	hypothetical protein	Unknown function
32*_II_*	+2.8	±0	A8YEX3_MICAE	hypothetical protein	Unknown function
32*_III_*	+3.9	±0	A8YEX3_MICAE	hypothetical protein	Unknown function
33	−2.2	+3.6	A8YM15_MICAE	hypothetical protein	Unknown function
34	+3.0	±0	A8YJU1_MICAE	hypothetical protein	Unknown function
35	±0	−2.0	A8YNN7_MICAE	similar to vanadium chloroperoxidase	Unknown function

1 See Fig. 1 for proteomic gel and spot numbers.

2 Accession numbers according to SWALL database.

3 Protein function according toTrEMBL database.

4 Different isoforms/fragments detected, n.q. not quantified.

PB: phycobiliproteines, PBL, phycobilisome linker proteines.

Some of the proteins affected by the loss of microcystin, including Calvin cycle enzymes, were previously shown to depend on the redox state of the cell and to interact with thioredoxins as electron carriers [17], [18]. In this context it is interesting to note that two NADPH-dependent reductases, glutathione reductase (Gor) and a thioredoxin reductase were also included among those differentially expressed proteins (Table 1). These data raised the possibility that loss of microcystins may affect redox-dependent processes. Further, reciprocal results obtained in the WT and mutant Δ*mcyB* with respect to relative abundances under the light/dark regime (Table 1) supported this notion.

### Microcystin binds to specific proteins of *Microcystis* via redox-sensitive cysteines

**Figure 2:**
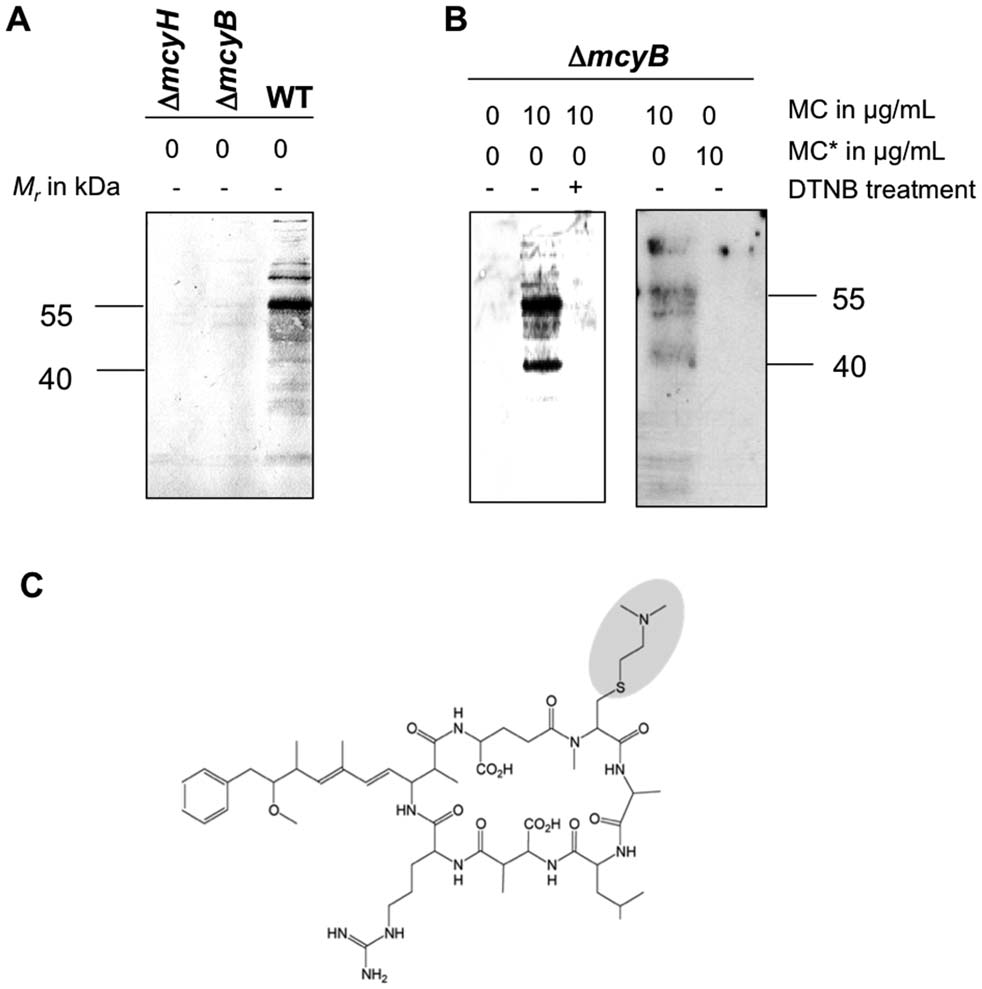
Immunoblot analyses of soluble *Microcystis* protein extracts using a microcystin-specific antibody. A) Protein extracts of the Δ*mcyH*, Δ*mcyB* mutants and wild type. B) Protein extracts of Δ*mcyB* mutant prior and after addition of microcystin-LR and DTNB pre-treatment (left). Protein extracts of Δ*mcyB* mutant with addition of microcystin-LR or cysteamin-linked microcystin-LR MC* (right). C) Modified microcystin variant with a cysteamin group attached to the N-methyldehydroalanine moiety.

In an attempt to investigate sub-cellular localization of microcystin at different light conditions, we applied a highly specific antibody, which is known to reliably quantify the toxin from environmental samples [19] for the detection of microcystin in protein fractions. In Western-blotting experiments we could detect several distinct bands in protein extracts from the wild type but not in the microcystin-deficient mutants Δ*mcyB* and Δ*mcyH* (Fig. 2A) suggesting specific binding of microcystin to certain proteins. The interaction between microcystin and the proteins was highly stable despite the denaturing conditions used for sample preparation and protein gel electrophoresis. The observed bands could thus unambiguously be assigned to microcystin being attached to proteins. The fact that the cyclic heptapeptide of about 1 kDa binds to the proteins could explain for the differences observed in protein isoform formation between the WT and the microcystin-deficient mutants in the proteomic experiment.

It has been shown that microcystin interacts with its eukaryotic protein phosphatase targets through its N-methyl-dehydroalanine moiety [3]. To clarify, whether a similar interaction occurs with proteins from *Microcystis*, microcystin was added to native protein extracts of the microcystin-deficient Δ*mcyB* mutant in concentrations that are typically detected in the wild-type strain (Fig. 2B). Using this *in vitro* approach, we could detect microcystin attached to proteins in similar pattern as found for the wild type *in vivo*. Again, one of the predominant bands showed a size of about 55 kDa. To verify whether microcystin binds to cysteines of these proteins, we used 5,5′ dithio-bis(2-nitrobenzoic acid) (DTNB) to block free SH-groups in the protein extracts from mutant Δ*mcyB*. This treatment severely inhibited binding of microcystin to proteins extracted from Δ*mcyB*. Further evidence for the nature of the microcystin-protein interactions came from experiments where we modified the vinyl group of the N-methyldehydroalanine moiety of microcystin with cysteamine (Fig. 2C). This modification abolished the covalent interaction to proteins in extracts of the Δ*mcyB* mutant (Fig. 2B). These experimental findings strongly suggest that microcystin forms stable thioether bonds with cysteines of a number of *Microcystis* proteins *in vivo* and *in vitro*.

### Microcystin binding partners show an altered accumulation in microcystin-deficient mutants

**Figure 3:**
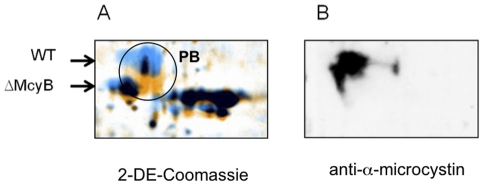
Comparative proteomic analysis of phycobiliproteins. A) Mass shift of phycobiliproteins (PB) in *Microcystis aeruginosa* PCC 7806 wild type (blue colouring) compared to microcystin-deficient Δ*mcyB* mutant (orange colouring) as seen by 2DE-analysis. Proteins with differential mass in wild type and mutant are encircled. B) Corresponding immunoblot analysis using the microcystin-specific antibody.

In order to identify the predominant microcystin-binding partners we performed an additional 2D gel electrophoresis experiment and visualized the microcystin-binding spots by parallel immuno-detection. The comparison of the signal distribution from the immunoblot with the parallel run Coomassie stained gel showed congruence for a number of spots, although a considerable part of signals had no visible counterparts in the Coomassie stained gel and obviously result from an interaction with proteins of lower abundance (data not shown). A strong signal was obtained with one of the large phycobiliprotein (PB) spots, which contained two proteins: the phycocyanin beta subunit CpcB and the allophycocyanin subunit ApcA. Interestingly, the same spot showed a mass shift of about 1 kDa in the Coomassie stained gels with soluble proteins from the wild type compared to the Δ*mcyB* mutant (Fig. 3). This mass shift could well correspond to microcystin (MW: 995) being attached to the protein(s). This example supports the assumption that differences in isoform formation between wild type and mutant strain could result from the covalent binding of microcystin to *Microcystis* proteins.

Another strong signal was obtained with a clearly visible protein spot of about 55 kDa that was identified as RbcL by mass spectrometry. RbcL is the large subunit of the universal carboxylating enzyme ribulose 1,5-bisphosphate carboxylase/oxygenase (RubisCO). Further spots were identified as the small subunit of RubisCO RbcS, phosphoribulokinase (Prk), glutathione reductase and two hypothetical proteins (Table 2). Interestingly, all microcystin binding proteins were also found to be differentially expressed in the previously performed proteomic study (Table 1) indicating that microcystin binding to proteins may at least partly explain the differences in the abundance of these proteins. As microcystin binds to cysteines of a number of proteins that are known to be redox-regulated in cyanobacteria [17], [20], we were thus following the idea whether the microcystin-binding could have a physiological relevance under these conditions.

**Table 2:** List of microcystin binding proteins in extracts of the wild type *M. aeruginosa* PCC 7806 (WT) which were identified by 2D electrophoresis and subsequent immunoblots against microcystin.

Spot no.^1^	Entry name^2^	Identity/Protein function^3^	Category	Differentially expressed in WT and Δ*mcy*B^4^
4	A8YF93_MICAE	small subunit of RubisCO	Photosynthesis	Yes
5	A8YF91_MICAE	large subunit of RubisCO	Photosynthesis	Yes
6	A8YD92_MICAE	phosphoribulokinase	Photosynthesis	Yes
10	A8YJM8_MICAE	phycocyanin alpha subunit	Photosynthesis	Yes
PB	A8YJM7_MICAE	phycocyanin beta subunit	Photosynthesis	n.q.
PB	A8YFC5_MICAE	allophycocyanin alpha subunit	Photosynthesis	n.q.
20	A8YMK3_MICAE	glutathione reductase	Biosynthesis of cofactors, prosthetic groups and carriers	Yes
33	A8YM15_MICAE	hypothetical protein	Hypothetical	Yes
35	A8YNN7_MICAE	similar to vanadium chloroperoxidase	Hypothetical	Yes

1 See Fig. 1 for proteomic gel and spot numbers.

2 Accession numbers according to SWALL database.

3 Protein function according to TrEMBL database.

4 See Table 1.

n.q. not quantified due to high amount and amagalmation.

### High light stimulates the microcystin binding to proteins

**Figure 4:**
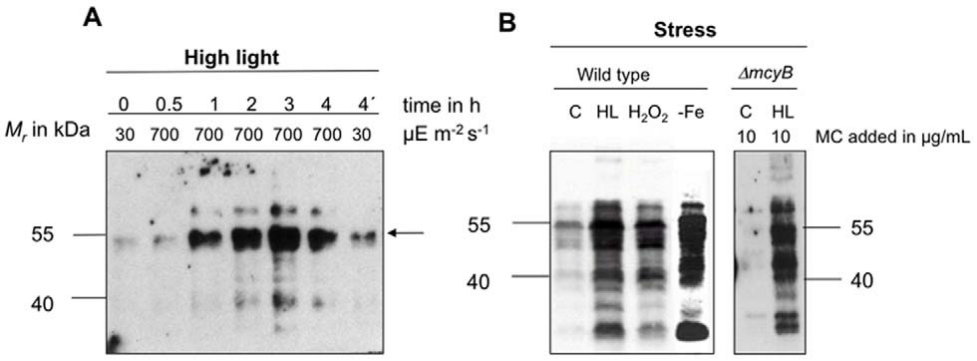
Impact of light and oxidative stress on microcystin binding to proteins. A) Immunoblot analyses of soluble *Microcystis* protein extracts with an anti-microcystin antibody after high light treatment of 700 µmol photons m^−2^ s^−1^ for up to 4 hours. The irradiation time of the individual samples is indicated. The sample 4′h was irradiated with high light for three hours and subsequently transferred to low light for one hour. B) Immunoblot analysis of a low light adapted culture (C) that was treated for 4 hours with 700 µmol photons m^−2^ s^−1^ high light (HL), 10 µM H_2_O_2_ or in parallel grown under low light in the absence of iron (-Fe). Immunoblot analysis of a low light adapted culture of the Δ*mcyB* mutant and a culture treated for three hours with high light (700 µmol photons m^−2^ s^−1^) after microcystin addition.

To this end, we examined the amount of microcystin bound to proteins under conditions known to affect the oxidation state of cysteines such as elevated light intensity. First, low light grown cells (30 µmol photons m^−2^ s^−1^) of the PCC 7806 wild-type strain were transferred to very high light of about 700 µmol photons m^−2^ s^−1^ and the amount of microcystin was assessed by immunoblot analyses. Already after one hour treatment we observed a remarkable increase of microcystin bound to proteins (Fig. 4A). The microcystin-binding was further enhanced after treatments for up to three hours. Similar results were obtained with four independent biological replicates and at other time points of the logarithmic growth phase (data not shown). One aliquot of the culture was transferred back into low light of 30 µmol photons m^−2^ s^−1^ for one hour after three hours treatment with high light (Fig. 4A, sample 4′). This treatment led to a marked decline in the amount of microcystin bound to proteins compared with those maintained under high light illumination for the entire four hours. It should be noted that even under conditions obviously favouring microcystin binding to proteins this interaction was limited to a specific subset of proteins indicating a specific interaction to defined targets. We have also evaluated whether the applied light treatment was causing oxidative stress in the cultures using chlorophyll-a bleaching and a lipid peroxidation assay. Both assays supported the assumption that the light conditions used were indeed causing oxidative stress (data not shown).

To proof whether or not other conditions inducing oxidative stress have a similar impact on microcystin binding to proteins, *Microcystis* cultures were exposed to 10 µM H_2_O_2_ for four hours or grown in the absence of iron (Fig. 4B). In both cases, the rise in microcystin-binding as well as the general binding pattern was similar to that found after high light treatments. These results supported the view that a stimulation of microcystin-binding to proteins is part of a general oxidative stress response in cells of *M. aeruginosa* PCC 7806 wild type. The attraction of higher amounts of microcystins by proteins might be caused by conformational changes of protein targets leading to an increased susceptibility of cysteines and not by changes in the availability of microcystin *in vivo*. In agreement with this hypothesis, an increase in microcystin binding to proteins of the Δ*mcyB* mutant was found *in vitro*, when proteins were obtained after exposure of mutant cells to high light of 700 µmol photons m^−2^ s^−1^ for three hours (Fig. 4B).

**Figure 5:**
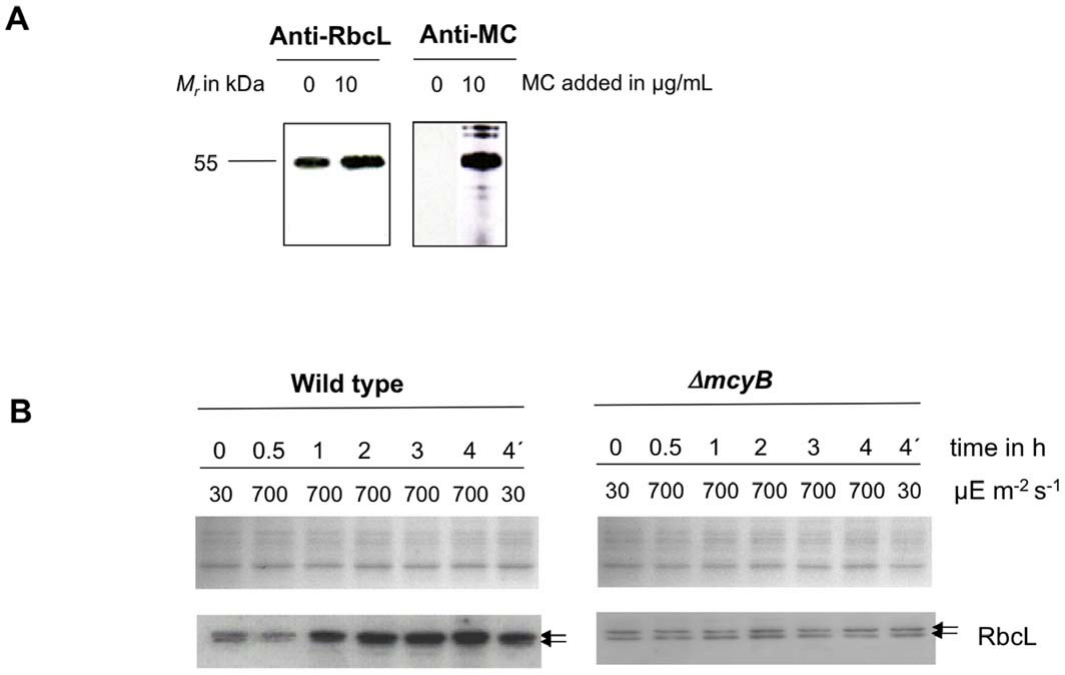
Quantitative analysis of RbcL. A) Immunoblot analysis of *in vitro* expressed and affinity purified RbcL protein using a RbcL-specific antibody and a microcystin-specific antibody, respectively. Microcystin was added as indicated. B) Representative Coomassie-stained gel pictures (upper panel) and immunoblot analyses (lower panel) with a RbcL-specific antibody of soluble protein extracts of *M. aeruginosa* PCC 7806 wild type (left) and the Δ*mcyB* mutant (right) after high light treatment of 700 µmol photons m^−2^ s^−1^ for up to four hours. The irradiation time of the individual samples is indicated. The sample 4′h was irradiated with high light for three hours and subsequently transferred to low light for one hour.

Among the many Calvin cycle enzymes found to be affected in the Δ*mcyB* mutant, we selected RbcL for a more detailed study. To test, whether microcystin can bind to the protein *in vitro*, we expressed the *rbcL* gene from *M. aeruginosa* PCC 7806 as a His-tag fusion protein in *E. coli* and purified the recombinant RbcL by Ni-agarose affinity chromatography under native conditions. Subsequently, the purified RbcL was incubated with 10 µg mL^−1^ microcystin and the resulting solution was analysed via denaturing SDS PAGE using antibodies against RbcL and microcystin, respectively. As a result, microcystin could clearly be detected attached to the RbcL protein in the parallel immunoblot experiments (Fig. 5A) suggesting that microcystin is able to bind to RbcL *in vitro*. We have further re-confirmed *in vivo* microcystin-binding to RbcL within a carboxysome-enriched fraction that contained high amounts of RbcL (Fig. S2).

RbcL was frequently found to be differentially expressed in wild type and microcystin-deficient mutants under various conditions tested (Table 1 and unpublished data). In order to test whether the differential accumulation is pronounced under conditions stimulating microcystin-binding, the RbcL levels were analyzed in protein samples collected during a time course of high light treatments (0.5 to 4 h) in the wild type and the *ΔmcyB* mutant. In all samples two bands of about 52–55 kDa were detected for RbcL. The presence of two RbcL isoforms could indicate high turnover rates of this protein in *Microcystis.* A similar pattern was reported from *Chlamydomonas* and discussed as degradation [21]. Samples of the wild type showed a clear increase in the amount of RbcL after 0.5 to one hour high light treatment suggesting an increased expression and/or stability of this protein (Fig. 5B). Moreover, it is obvious that especially the high molecular band of 55 kDa increased in its amount, which indicates a possible delay in RbcL turnover. In contrast, no corresponding increase was observed in cell extracts from any of the mutant cultures suggesting that the expression or stability of RbcL in the microcystin-deficient mutant Δ*mcyB* is not altered under high light conditions.

### RbcL shows a higher sensitivity to subtilisin in the absence of microcystin

In order to prove whether the higher accumulation of intact RbcL in the wild type is due to higher protein stability caused by conformational changes, we performed a protease assay based on similar experiments with RbcL from *Chlamydomonas.* In these experiments RbcL was highly sensitive against subtilisin degradation and, moreover, the degradation pattern was clearly dependent on the redox state of cysteines [21]. Similar redox-dependent alterations seem to occur with RbcL from *Microcystis*. We have compared the oxidation state of RbcL cysteines in wild-type and mutant strain via treatment of sulfhydryl groups with iodoacetate and iodoacetamide, respectively [22]. As a result we could find clear differences in the alkylation pattern of RbcL in dependence of the presence or absence of microcystin (Fig. S3).

**Figure 6:**
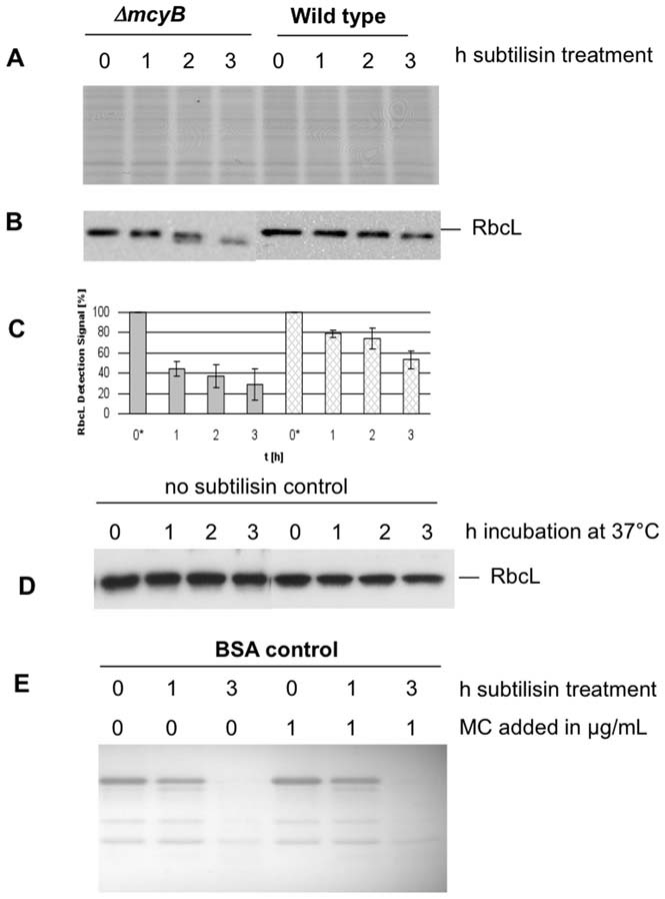
Protein degradation assay. A) Coomassie gel picture showing *M. aeruginosa* PCC7806 wild type and Δ*mcyB* cell extracts treated for up to 3 hours with 0.2 µg/mL subtilisin. B) Immunoblot analysis using a specific RbcL antibody. C) Quantification of RbcL signal intensity from three independent subtilisin assays for *M. aeruginosa* PCC7806 wild type and Δ*mcyB* mutant. * The starting points were adjusted to 100% D) Control treatment without addition of subtilisin for three hours. E) Control digestion of BSA with subtilisin in presence or absence of microcystin.

In a next step, wild-type and mutant protein extracts isolated from cultures exposed to high light conditions of 700 µmol photons m^−2^ s^−1^ for three hours were treated with subtilisin (Fig. 6). RbcL was rapidly degraded by subtilisin in samples of the mutant, while the RbcL degradation was clearly delayed in wild-type extracts with bound microcystin (Fig. 6B and C). To verify that the observed degradation is indeed correlated with the subtilisin treatment and not due to a different expression of proteases in wild type and mutant, in a control experiment samples were incubated without subtilisin, which did not result in significant changes in the amount of RbcL (Fig. 6D). Prior to the protease treatment wild-type and mutant extracts were purified using desalting columns to remove free microcystin. In order to exclude a possible inhibition effect of microcystin on subtilisin, an additional control experiment was performed where BSA was treated as control protein with subtilisin in presence or absence of microcystin showing no difference in subtilisin activity (Fig. 6E). We therefore conclude that RbcL in the microcystin-free mutant *ΔmcyB* indeed shows a higher susceptibility to subtilisin degradation. The observed differences in RbcL accumulation after high light treatment may thus at least partly be due to a delay in degradation of the protein caused by conformational differences in the wild type. Transcriptional differences may further contribute to the observed changes in RbcL accumulation. The aim of the present study, however, was to assess the impact of protein-bound microcystin.

### The Δ*mcyB* mutant is more sensitive to oxidative stress conditions

**Figure 7:**
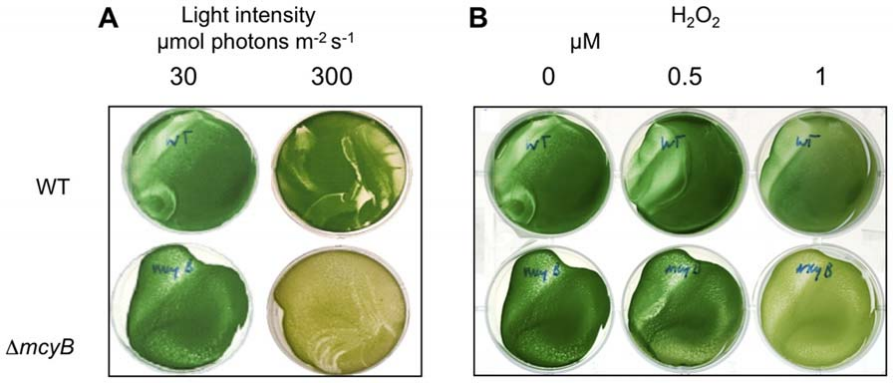
Photographs of *M. aeruginosa* PCC 7806 wild type (WT) and Δ*mcyB* mutant. A)grown on 6-well plates for five days under low light (30 µmol photons m^−2^ s^−1^) or high light (300 µmol photons m^−2^ s^−1^) conditions, B)grown on 6-well plates for five days under low light conditions with increasing concentrations of H_2_O_2_ supplemented to the medium.

If microcystin affects the stability or activity of key enzymes of the Calvin cycle such as RbcL, RbcS and Prk, one should expect differences in the growth behaviour between microcystin-containing and microcystin-free cells, particularly under conditions known to affect the oxidation state of cysteines. To examine this possibility, *Microcystis* PCC 7806 wild type and mutant Δ*mcyB* were grown on parallel plates under both low light and high light conditions. In agreement with our earlier studies, wild type and mutant did not show any difference in growth under low light conditions (30 µmol photons m^−2^ s^−1^) (Fig. 7). However, in a number of replicate experiments the mutant showed a clearly higher sensitivity to high light of about 300 µmol photons m^−2^ s^−1^. Whereas the wild type remained green, the mutant typically bleached out after three to four days of cultivation under permanent high light. In addition, wild type cells showed a better recovery after bleaching when exposed to 300 µmol photons m^−2^ s^−1^ for an entire week and subsequently transferred back to low light conditions of 30 µmol photons m^−2^ s^−1^ in liquid culture (Fig. S4). Additionally, we searched for similar phenotypic consequences under other conditions triggering oxidative stress. To this end, wild type and mutant cultures were treated with H_2_O_2_ under low light conditions. As expected, the microcystin-deficient mutant was more susceptible to 1 µM H_2_O_2_ (Fig. 7). Taken together, these phenotypic changes well support a physiological role of microcystin under high light and oxidative stress conditions in *Microcystis* cells producing the peptide.

## Discussion

There is no doubt that microcystins belong to the most potent toxins in aquatic environments. However, this “toxic power” does not necessarily reflect the primary function of microcystins for its producing cell; in particular as critical amounts are typically not released into the water body by exponentially growing cells. There is a general concern whether microbial antibiotics do play a major role in defence in their respective ecosystems, instead there is more and more evidence for principal roles of small molecules in cell-cell communication rather than in antibiosis [23]. The results of this study indicate an entirely new function of a secondary metabolite and a surprisingly close connection of microcystin to the primary metabolism of cyanobacteria.

Binding of microcystin to cellular components has been postulated earlier. Jüttner and Lüthi observed a binding of the toxin to cyanobacterial antennae proteins [24], while Vela and coworkers reported the association of microcystin with a set of proteins *in vitro* and *in vivo* [25]. However, in these studies the binding was considered as unspecific and non-covalent, and not linked to a physiological function. Our data reveal a specific and covalent interaction of microcystin with a subset of proteins resulting in their altered accumulation under different light or redox conditions. These different findings, however, do not necessarily contradict each other. In order to establish a covalent bond, microcystin has to build up a non-covalent interaction first. Under high light and oxidative stress conditions, this non-covalent interaction is then strengthened by covalent interactions of cysteines and the N-methyldehydroalanine position of microcystin. Though we are far from understanding the role(s) of microcystin, an increasing number of facts indicate an intracellular function of microcystin related to oxidative stress: (1) the increased transcription of *mcy* mRNA under high light and iron-deficient conditions [13], [14]; (2) the remarkable overlap of proteins affected by the loss of microcystin with known redox-sensitive proteins in cyanobacteria; (3) the binding of microcystin to cysteines that are sensitive to redox changes; 4) the strong increase of microcystin binding to proteins under high light and oxidative stress conditions; and 5) the increased sensitivity of microcystin-deficient mutants under high light and oxidative stress conditions.

The observed microcystin binding to specific proteins in cells of the natural producer *Microcystis* could possibly precede dimerization of cysteines and thus detain enzymes from conformational changes or even loss of their catalytic activity. Remarkably, the Calvin cycle is represented by several of its enzymes. The list of individual proteins almost completely overlaps with the list of known thioredoxin interaction partners of the Calvin cycle [17], [18]. With RbcL, RbcS and Prk at least three of the proteins were shown to directly interact with microcystin (see Table 2). RbcL is known as a “cysteine sensitive” protein on the basis of its inactivation by thiol-directed reagents such as p-chloromercuribenzoate [26]. For chloroplast Prk it could be shown that formation of a disulfide bond can even physically block the enzyme active site [27]. The CP12 protein which was also found to be differentially expressed in wild type and mutant cells shows a redox-dependent binding to Calvin cycle enzymes in cyanobacteria as well as in chloroplasts [16]. With glutathione reductase, another microcystin binding partner fits well into the general redox context. Future studies have to show the individual impact of microcystin on the expression, stability and activity of as many *Microcystis* proteins as possible. This will allow us to integrate microcystin into the bioenergetic context and to interpret the phenotype of microcystin-deficient mutants under high light and conditions triggering oxidative stress.

A role of microcystin as protein-modulating metabolite and protectant against oxidative stress as suggested in our study could be the primary and original function of this secondary metabolite which would explain why probably all ancestral cyanobacteria produced these heptapeptides [7]. The selective loss of the biosynthetic genes could be related to the enormous costs that are associated with the maintenance and activity of the giant gene cluster and the corresponding enzymatic complex. Possibly, the loss of microcystin biosynthesis was compensated by the evolution of other traits for the protection against oxidative stress. Considering RbcL as a major target, its enclosure in carboxysomes during the later cyanobacterial radiation could have made some of the protective mechanisms against oxidative stress dispensable. Still, microcystin production is very widespread among highly diverse bloom-forming species of cyanobacteria. The retention of microcystin in this environmentally successful group of cyanobacteria could thus relate to the specific lifestyle and the niche adaptation of these cyanobacteria, since in the bloom situation they are exposed to extremely high irradiances and oxygen over-saturation.

The data obtained in this study immediately raise the question whether non-toxic *Microcystis* strains have evolved other mechanisms to compensate for the lack of microcystin or if these strains have indeed disadvantages under conditions triggering ROS development. However, if we compare the different strains we have to consider both the high costs of microcystin production and the potential benefit as postulated in this study. Recent studies comparing toxic and non-toxic *Planktothrix* strains revealed that microcystin producing strains were clearly winning out against non-toxic strains when environmental conditions were limiting growth [28]. Although this study supports a general impact of microcystin on the fitness of the producing cyanobacteria, it did not include conditions causing oxidative stress. Remarkably, this and other studies observed a decrease in microcystin quota per cell in the late exponential growth phase. In the light of the findings of the present study this decrease could be related to an increase in microcystin binding to proteins in senescent cultures that are accumulating ROS. In another study, Kardinaal and co-workers have looked at the competition for light between toxic and nontoxic strains of *Microcystis*. Here, the toxic strains were in disadvantage; however, the light conditions used were not in the range typically causing oxidative stress [29]. In order to translate the findings in our study to field situations thus thorough competition experiments are needed that compare the fitness of wild type and microcystin deficient mutant strains under oxidative stress conditions.

## Materials and Methods

### Cultivation of strains

The strain *M. aeruginosa* PCC 7806 was kindly provided by R. Rippka (Institute Pasteur, France). Mutants of this strain unable to produce microcystin were obtained by insertion of a chloramphenicol (Cm)-resistance cartridge in the genes *mcyB* and *mcyH*, respectively [30], [31]. Wild-type and mutant cultures were grown at 23°C in batch cultures with BG11 medium [32]. For the light experiments three independent cultures were grown under 16 µmol photons m^−2^ s^−1^ and then exposed to high light conditions of 70, 300, or 700 µmol photons m^−2^ s^−1^ for up to two or four hours. Light intensities were measured using a Li-Cor LI250 light meter (Walz, Effeltrich, Germany). Iron deficiency was achieved by washing and transfer of the cultures into iron-free BG11 medium. Iron depletion was confirmed by monitoring the blue-shift of the chlorophyll absorption at 680 nm.

### Protein isolation and immunoblot analysis

In order to isolate the soluble proteome of *Microcystis*, cells were harvested by centrifugation, washed and resuspended in 10 mM Tris-HCl (pH 7.8) containing 1 mM EDTA and 1 mM PMSF as described in [10]. Cells were disrupted by French Press (900 lb/in^2^ at 4°C). Soluble and membrane proteins were further separated by ultracentrifugation (20,000×g for 45 min at 4°C). 10 µg of total protein extracts were treated with β-Mercaptoethanol (14.4 mM) and SDS (2%) containing loading buffer and heated at 100°C for 5 minutes. Subsequently, proteins were separated using 12.5% Tris-glycine SDS-PAGE and blotted onto nitrocellulose filters (Hybond^TM^-C Extra, Amersham). Filters were blocked overnight with 5% skim milk powder in PBS-T (0.1% TWEEN 20) and probed with anti-microcystin-antibody (Alexis, Farmingdale, USA, 1∶10000, 1 h) or anti-RbcL antibody (Agrisera, 1∶50000, 1 h) and a secondary antibody (Sigma, HRP conjugated secondary antibody, 1∶50000, 1 h) in PBS-T containing 2% low fat milk powder. Antibody incubations were followed by washings in PBS-T. All steps were performed at 4°C with agitation. Detection of CP12, ATP synthase and FtsZ cell division protein were performed with specific polyclonal antibodies against the respective polypeptides as described previously [16], [33], [34]. Signal development was achieved using the SuperSignal West Pico Chemoluminescent Substrate Kit (Pierce, Rockford, USA) according to the standard protocol.

### Preparation of 2-dimethylaminoethanethiol-microcystin LR

All solvents used for reaction were placed in nitrogen in order to protect free thiol groups. Microcystin LR (5.0 mg in 100 µl methanol) was mixed with DMSO (200 µl), 2-dimethylaminoethanethiol hydrochloride (7.0 mg in 200 µl H_2_O), and 3 µl of 500 mM EDTA (pH 8.0). After stirring for one hour at 50°C, 10 ml of H_2_O was added to reaction mixture, and this mixture was subjected to ODS SPE column (0.5 mg, Chromabond C18, Macherey-Nagel, Düren, Germany). Methanol eluted fraction was subjected to RP-HPLC (Nucleosil 5C18, 21×250 mm, Macherey-Nagel, Düren, Germany) applying the following gradient of solventA/B 40∶60 for 10 min, to 100/0 in 60 min (solventA: 83% acetonitrile, solventB: H_2_O containing 0.1% TFA), at 238 nm, and at a flow rate 10 ml min^−1^ to yield 2-dimethylaminoethanethiol-microcystin LR (4.8 mg).

### 2D electrophoresis

2-DE of the soluble protein fraction was carried out as described in [35]. The lyophilized extracts (400 µg protein) were dissolved in rehydration solution (8 M urea, 2 M thiourea, 19.4 mM DTT, 1% CHAPS, 0.5% carrier ampholytes (Pharmalyte, pH 3–10, Amersham Bioscience) and traces of BPB) and were separated in the first dimension on Immobilized Dry Strip Gels, pH 4–7/180 mm (Amersham Bioscience). The second dimension was carried out on SDS-containing polyacrylamide gels (12.5% acrylamide, 230×230×0.75 mm). A molecular mass marker (broad range, BioRad) was separated in the same gel. For Coomassie-staining, gels were treated for 30 min in staining solution (50% methanol, 10% acetic acid, 0.1% CBB R250) and destained with 20% methanol/10% acetic acid. Gels were washed with double distilled water for 5–10 min and scanned for further computational analysis. At least eight gels were performed per condition (six gels from the respective biological replica and one technical replica of two of the biological replica). Finally, eight well resolved gels of one condition were grouped, warped and fused to one gel via Delta2D version 4.0 (DECODON, Greifswald, Germany). Differences in the relative spot intensity of the four fusion gels were determined for comparative proteomic analysis of wild type and Δ*mcyB* mutant under light and dark conditions (Delta2D). Protein spots whose expression changed more than two-fold were excised from the Coomassie-stained gel manually using pipette tips that were cut to form an orifice of approx. 1.5 mm inner diameter.

### Mass spectrometric analysis

Protein spots were digested with trypsin, the resulting peptide-containing solution was prepared onto a MALDI target (384/600 µm AnchorChip™, Bruker Daltonik, Bremen, Germany) and the peptide masses were measured by MALDI-TOF-MS using a Reflex III mass spectrometer (Bruker Daltonik) as described [35]. Proteins were initially identified by searching a protein database of *M. aeruginosa* PCC 7806 (EMBL accession numbers AM778843 to AM778958) using the MASCOT software (Matrix Science, London, UK) *via* BioTools 3.0 software (Bruker Daltonik). A mass tolerance of 80 ppm and 1 missing cleavage site were allowed, oxidation of methionine residues was considered as variable modification, and carbamidomethylation of cysteines as fixed modification. In consideration of the fact that searching a small database increases the risk of false positive identifications the searches were repeated against the SWALL database using the taxonomy filter Eubacteria (Swiss-Prot and TrEMBL, releases 57.14 and 40.14 containing 322617 and 6304203 eubacterial sequences, respectively).

### RbcL expression and *in vitro* binding analysis

For *in vitro* expression of RbcL the corresponding gene, amplified from PCC 7806 (RbcL_NdeI.fw: CATATGGTGCAAGCCAAATCC, RbcL_BamHI.rv: GGATCCGAGGGTATCCATAGCCTC), were cloned into the pET15b vector system (Novagen). Thus, the His-tagged protein was isolated via nickel-agarose under native conditions. Purity was successfully checked by SDS-PAGE analysis of respective elution fractions (data not shown). For the subsequent binding analysis the recombinant protein (approximately 100 ng). For the subsequent binding analysis the recombinant protein was concentrated in buffer containing 50 mM HEPES, pH 8.0, 40 mM NaHCO_3_ and 20 mM MgCl_2_ via dialysis. In order to check the putative binding capacity of MC to RubisCO the protein sample was preincubated at 30°C for 30 min. Finally the sample was split into two vials and microcystin-LR was added to one of the samples in a final concentration of 10 mg/L^−1^. Both samples were incubated at 30°C for one hour and then further treated for 30 min by the addition of DTT (5 mM). All fractions were analysed by SDS-PAGE and immunoblotting, respectively.

### Alkylation of cysteine sulfhydryl groups

To determine the oxidation state of cysteines they were differentially modified using a method described by Creighton [22]. The RbcL-enriched protein fractions were transferred from extraction buffer into assay buffer using Amicon ultra 30K (Millipore) in three steps to wash off smaller proteins and peptides. First, the buffer only contained 10 mM Tris-HCl (pH 8.0) plus 1 mM EDTA. Subsequently, the extracts were transfered into buffer including 8 M urea and finally into the complete assay buffer including all of the former plus 10 mM DTT (Unfolding-activation buffer: 10 mM TrisHCl (pH 8.0), 8 M urea, 10 mM DTT). The protein solutions were incubated at 37°C for 30 minutes to guarantee complete unfolding of the protein and reduction of thiols and disulfide bonds to active sulfhydryl groups. For competitive alkylation of thiol groups, aliquots of 40* µ*L were mixed with 10* µ*l of either iodacetamide- or iodacetate stock (Solution A: 46.5 mg iodacetamide in 1 ml 0.25 M Tris-HCl (pH 8.0); solution B: 0.5 mg iodacetic acid in 1 ml 0.25 M Tris-HCl (pH 8.0).The mixtures were incubated at RT for 15 minutes and kept on ice until native electrophoresis.

### Protease assay

The RubisCO degradation assay was carried out using the serine protease subtilisin (Sigma-Aldrich, Munich, Germany). Wild type and mutant proteins from were transferred into activation buffer usually used for measuring RubisCO activity [36] using Amicon® Ultra centrifugal filters 30K (Millipore™, Ireland) to guarantee equal reaction milieu for each replicate. The transfer of the protein into buffer is of particular importance, because the activity of subtilisin highly depends on the pH. An extra benefit of exchanging the buffer is to exclude unbound microcystin, hence eliminating possible inhibitory effects of free microcystin to subtilisin. The protein concentration was adjusted to 1 mg/ml. To check whether microcystin had any inhibitory effects on the protease activity of subtilisin itself, 2* µ*g/ml microcystin were added to separate aliquots from Δ*mcyB* extracts. Volumes of 120* µ*L from each sample were set up for the degradation assay on a thermo shaker at 30°C. The degradation was started by adding subtilisin to a final concentration of 0.2* µ*g/ml (ratio 1* µ*g subtilisin: 5000* µ*g protein) and the samples were incubated for up to 3 hours. Protein degradation was stopped by addition of 10 mM PMSF and keeping the probes refrigerated until denaturisation in SDS-PAGE loading buffer at 95°C for 10 minutes. Additionally, a control degradation assay using BSA was performed. 0.1 mg/ml BSA was incubated in activation buffer together with 2* µ*g/ml microcystin for 1 hour at 30°C before adding subtilisin to a final concentration of 1* µ*g/ml and running the degradation assay as described above.

## Supporting Information

Table S1 List of identified proteins by PMF with detailed information to *p*I and molecular mass (comparison of deduced data from the *Microcystis* database with apparent data from the 2D analysis) as well as mowse score, number of matching peptides and sequence coverage by matched peptides.
(DOC)

Figure S1 Immunoblot analysis of soluble fraction of *Microcystis aeruginosa* PCC 7806 wildtype (wt) and microcystin-deficient mutant Δ*mcy*B after exposition to dark condition for 2 hours. Blots were obtained after separation (15% SDS-PAGE) and transfer of soluble protein extracts (40 µg) to nitrocellulose membranes. Equality of the samples was controlled by Ponceau staining of the membrane as well as Coomassie staining of the duplicated gel. Immunoblot analysis was performed for selected differential protein spots with antibodies against ATP synthase, CP12 like polypeptide, FtsZ (used as loading control) and microcystin, respectively.
(JPG)

Figure S2 Microcystin-binding in carboxysome-enriched fraction that was obtained by fractionated solubilization. A) Coomassie stained fractions of wild type and Δ*mcyB* mutant. B) Immunoblot analysis using anti-RbcL antibody (identity of RbcL was further confirmed using mass spectrometry). C) Immunoblot analysis using anti-microcystin antibody.
(JPG)

Figure S3 Differential alkylation pattern of RbcL of PCC 7806 wild type and ΔmcyB mutant, respectively, with iodoacetamide and iodoacetate leading to charge differences depending on the presence of free thiol groups. RbcL fractions were run on native PAGE and subsequently evaluated by immunoblot analysis with an RbcL specific antibody.
(JPG)

Figure S4 Recovery of Δ*mcyB* mutant and wild type after one week irradiation with 300 µmol photons m^−2^ s^−1^ and subsequent transfer to 30 µmol photons m^−2^ s^−1^ for three days. NT, non-treated control of wild type; C, control treated for one week with 300 µmol photons m^−2^ s^−1^ without recovery of wild type.
(JPG)
