# Supplementary material for: The Cyanobacterial Hepatotoxin Microcystin Binds to Proteins and Increases the Fitness of Microcystis under Oxidative Stress Conditions
Source: PLoS One. 2011 Mar 18;6(3):e17615. doi: 10.1371/journal.pone.0017615 (PMC3060824; doi:10.1371/journal.pone.0017615)
Supplement: Table S1 — List of identified proteins by PMF with detailed information to pI and molecular mass (comparison of deduced data from the Microcystis database with apparent data from the 2D analysis) as well as mowse score, number of matching peptides and sequence coverage by matched peptides. (DOC) [file pone.0017615.s001.doc]

**Table S1**: List of identified proteins by PMF with detailed information to *p*I and molecular mass (comparison of deduced data from the *Microcystis* database with apparent data from the 2D analysis) as well as mowse score, number of matching peptides and sequence coverage by matched peptides

| **spot no.^1^** | **ORF^2^** | **gene** | **Identity^3^** | **category** | **Deduced *p*I** | **Apparent *p*I** | **Deduced mass in kDa** | **Apparent mass in kDa** | **score** | **coverage** | **match** | **comment** |
| --- | --- | --- | --- | --- | --- | --- | --- | --- | --- | --- | --- | --- |
| 1*_I-II_* | A8YJ50_MICAE |  | CBS domain containing membrane protein, CP12 like polypeptide | Photosynthesis | 5.39 | 5.5  5.8 | 23.142 | 21 | 131  125 | 43  35 | 10  8 | *p*I isoforms detected (2) |
| 2 | A8YBT0_MICAE |  | CBS domain containing membrane protein. CP12 like polypeptide |  | 5.27 | 5.8 | 22.810 | 23 | 95 | 38 | 6 |  |
| 3*_I-III_* | A8YNI2_MICAE | *tkt*A | Transketolase |  | 5.31 | 5.4  5.5  5.6 | 72.731 | 70 | 120  152  101 | 20  23  20 | 12  14  12 | *p*I isoforms detected (3) |
| 4*_I-II_* | A8YF93_MICAE | *rbc*S | small subunit of RubisCO |  | 7.59 | 6.4  6.6 | 13.316 | 13 | 94  76 | 63  46 | 6  5 | *p*I isoforms detected (2) |
| 5 | A8YF91_MICAE | *rbc*L | large subunit of RubisCO |  | 6.45 | 6.3 | 52.543 | 52 | 107 | 53 | 32 |  |
| 6*_I-II_* | A8YD92_MICAE | *prk* | Phosphoribulokinase |  | 5.13 | 5.3  5.4 | 38.036 | 40 | 141  91 | 32  21 | 10  7 | *p*I isoforms detected (2) |
| 7 *_I-II_* | A8YJ16_MICAE | *fba*A | fructose-bisphosphate aldolase class II |  | 5.31 | 5.6  5.7 | 39.156 | 43 | 148  131 | 30  21 | 11  8 | *p*I isoforms detected (2) |
| 8 *_I-IV_* | A8YJZ4_MICAE | *fbp*I | fructose-1,6-/sedoheptulose-1,7-bisphosphatase |  | 5.11 | 5.3  5.3  5.4  5.4 | 37.663 | 43  45  43  45 | 134  111  107  56 | 24  24  24  18 | 14  12  10  8 | *p*I isoforms detected (2), protein in different sizes detected (2) |
| 9 | [A8YF87_MICAE](http://rack7/mascot/cgi/protein_view.pl?file=../data/20060315/F088140.dat&hit=2) | *ccm*K | carbon dioxide concentrating mechanism protein CcmK |  | 7.73 | 6.3 | 12.080 | 12 | 94 | 61 | 5 |  |
| 10*_I-III_* | [A8YJM8_MICAE](http://rack7/mascot/cgi/protein_view.pl?file=../data/20060315/F088149.dat&hit=2) | *cpc*A | phycocyanin alpha subunit |  | 5.77 | 6.2  6.2  5.5 | 17.619 | 17  16  30 | 136  62  123 | 66  38  57 | 9  5  8 | *p*I isoforms detected (3), protein in different sizes detected (3) |
| 11*_various_* | A8YJM9_MICAE | *cpc*I | phycobilisome 32.1 kDa linker polypeptide, phycocyanin-associated, rod1 |  | 9.59 | From 5.0 till about 7 | 32.250 | From 12 till 35 | 105  103  121  115  115  114  68  93  56  60  109  93  60 | 27  23  27  29  27  27  21  23  15  17  35  25  24 | 9  7  8  11  7  9  6  7  4  6  7  7  7 | *p*I isoforms detected (13), protein in different sizes detected (13) |
| 12 | A8YD60_MICAE | *atp*A | ATP synthase CF1 alpha chain AtpA |  | 5.02 | 6.5_N-ter_ | 54.116 | 45_N-ter_ | 105_N-ter_ | 21_N-ter_ | 11_N-ter_ | protein fragment detected (N-terminal) |
| 13 *_I-II_* | A8YHP0_MICAE | *atp*B^4^ | ATP synthase CF1 beta subunit AtpB |  | 4.68 | 4.9  5.0 | 51.695 | 60 | 227  204 | 48  36 | 17  14 | *p*I isoforms detected (2) |
| 14 | A8YJB2_MICAE |  | thioredoxin reductase |  | 5.43 | 5.8 | 49.837 | 50 | 125 | 24 | 9 |  |
| 15 | A8YIW2_MICAE | *tuf* | translation elongation factor EF-Tu | Protein biosynthesis | 5.10  4.54 | 5.4  5.2_C-ter_  5.5_C-ter_ | 44.833  30.328 | 45  30_C-ter_  29_C-ter_ | 196  110_C-ter_  59_C-ter_ | 45  25_C-ter_  22_C-ter_ | 15  10_C-ter_  7_C-ter_ | *p*I isoforms detected (3); protein fragments detected (C-terminal) |
| 16 | A8YLU5_MICAE | *bcp* | Peroxiredoxin | Cellular processes | 4.61 | 4.7 | 22.046 | 15 | 98 | 31 | 5 |  |
| 36 | A8YCI8_MICAE | *sod*B | superoxide dismutase |  | 5.09 | 5.2 | 21.917 | 21 | 58 | 22 | 4 |  |
| 17 | A8YF09_MICAE | *glg*C | glucose-1-phosphate adenylyltransferase | Central intermediary metabolism | 6.68 | 6.5 | 48.652 | 47 | 269 | 44 | 20 |  |
| 18 | A8YBS4_MICAE | *cys*K | cysteine synthase | Amino acid biosynthesis | 5.31 | 5.7 | 34.405 | 36 | 125 | 41 | 9 |  |
| 19 | A8YC37_MICAE | *ilv*C | ketol-acid reductoisomerase |  | 5.38 | 5.8 | 36.111 | 37 | 188 | 45 | 13 |  |
| 20 | A8YMK3_MICAE | *gor* | glutathione reductase | Biosynthesis of cofactors, prosthetic groups and carriers | 5.66 | 6.2 | 49.379 | 50 | 79 | 19 | 9 |  |
| 21 | A8YMU9_MICAE | *hem*E | uroporphyrinogen decarboxylase |  | 5.62 | 5.9 | 39.222 | 40 | 144 | 36 | 10 |  |
| 37 | A8YJ48_MICAE | *trx*A | Thioredoxin |  | 5.02 | 5.3 | 12.376 | 13 | 108 | 60 | 6 |  |
| 22 *_I-II_* | A8YEP8_MICAE | *ahc*Y | adenosylhomocysteinase | Energy metabolism | 5.36 | 5.7  5.8 | 46.247 | 47 | 168  215 | 34  40 | 13  16 | *p*I isoforms detected (2) |
| 23 | A8YF09_MICAE | *pgi* | glucose-6-phosphate isomerase |  | 5.81 | 6.2 | 58.112 | 60 | 239 | 36 | 16 |  |
| 38 *_I-II_* | A8YF14_MICAE | *pgk* | phosphoglycerate kinase |  | 5.01 | 5.2  5.3 | 42.811 | 41 | 108  69 | 27  28 | 8  8 | *p*I isoforms detected (2) |
| 24 | [A8YJ06_MICAE](http://rack7/mascot/cgi/protein_view.pl?file=../data/20060315/F088171.dat&hit=2) | *fab*G2 | 3-oxoacyl-[acyl carrier protein] reductase; FabG2 | [Fatty acid, phospholipid and sterol metabolism](http://genome.kazusa.or.jp/cyanobase/Synechocystis/genes/category?class=G&subclass=G) | 5.59 | 6.0 | 25.288 | 23 | 117 | 48 | 10 |  |
| 25 | A8YJ05_MICAE | *thl* | acetoacetyl-CoA thiolase |  | 5.66 | 6.1 | 41.396 | 42 | 109 | 26 | 9 |  |
| 39 | A8YIV8_MICAE | *fab*I | enoyl-[acyl-carrier-protein] reductase |  | 5.31 | 5.6 | 27.779 | 30 | 114 | 39 | 11 |  |
| 26 | A8YFM0_MICAE |  | probable oxidoreductase | Hypothetical | 5.19 | 5.5 | 38.029 | 40 | 134 | 32 | 10 |  |
| 27*_I-IV_* | A8YG81_MICAE | *ocp* | water-soluble carotenoid protein |  | 4.81 | 4.8  4.8  5.0  5.0 | 35.729 | 40  35  40  35 | 113  110  180  144 | 25  28  42  36 | 9  10  15  11 | *p*I isoforms detected (2), protein in different sizes detected (2) |
| 28 *_I-II_* | A8YM76_MICAE |  | hypothetical protein |  | 5.45 | 5.7  5.8 | 69.069 | 68 | 132  168 | 22  29 | 10  14 | *p*I isoforms detected (2) |
| 29 | A8YDB5_MICAE | *mrp*A | hypothetical protein, MrpA |  | 4.95 | 5.4 | 35.999 | 38 | 145 | 43 | 10 |  |
| 30 | A8YHX5_MICAE | *mrp*C | hypothetical protein, MrpC |  | 4.2 | 4.1 | 15.5 | 17 | Identified via PSD (Try digestion: *m/z* 949.5 and 1077.5; Asp-N digestion: *m/z* 2020.1) | | |  |
| 31 *_I-II_* | A8YDG4_MICAE | *lys* | probable lysozyme |  | 6.51 | 5.7  6.0 | 21.285 | 24 | 163  89 | 41  29 | 10  6 | *p*I isoforms detected (2) |
| 32 *_I-III_* | A8YEX3_MICAE |  | hypothetical protein |  | 5.49 | 5.2  5.3  5.4 | 71.356 | 65 | 186  207  185 | 21  25  22 | 13  15  14 | *p*I isoforms detected (3) |
| 33 | A8YM15_MICAE |  | hypothetical protein |  | 5.29 | 5.4 | 31.055 | 31 | 118 | 34 | 9 |  |
| 34 | [A8YJU1_MICAE](http://rack7/mascot/cgi/protein_view.pl?file=../data/20060315/F088170.dat&hit=2) |  | hypothetical protein |  | 5.92 | 5.9 | 22.876 | 22 | 105 | 39 | 7 |  |
| 35 | A8YNN7_MICAE |  | similar to vanadium chloroperoxidase |  | 6.1 | 6.5 | 45.360 | 45 | 237 | 55 | 20 |  |
| PB | A8YJM7_MICAE  A8YFC5_MICAE | cpcB  apcA | phycocyanin beta subunit  allophycocyanin alpha subunit |  | 5.12  4.74 | 5.1 | 18.324  17.409 | 18 | 116  75 | 63  44 | 14  7 | spot contained  two proteins |

^1^ Spot-numbering according to Table 1

^2^ ORF designations according to SWALL database

^3^ Protein function according to TrEMBL databases.

^4^ Annotated as AtpD in SWALL database
