# Supplementary figures and images for: The Cyanobacterial Hepatotoxin Microcystin Binds to Proteins and Increases the Fitness of Microcystis under Oxidative Stress Conditions
Source: PLoS One. 2011 Mar 18;6(3):e17615. doi: 10.1371/journal.pone.0017615 (PMC3060824; doi:10.1371/journal.pone.0017615)

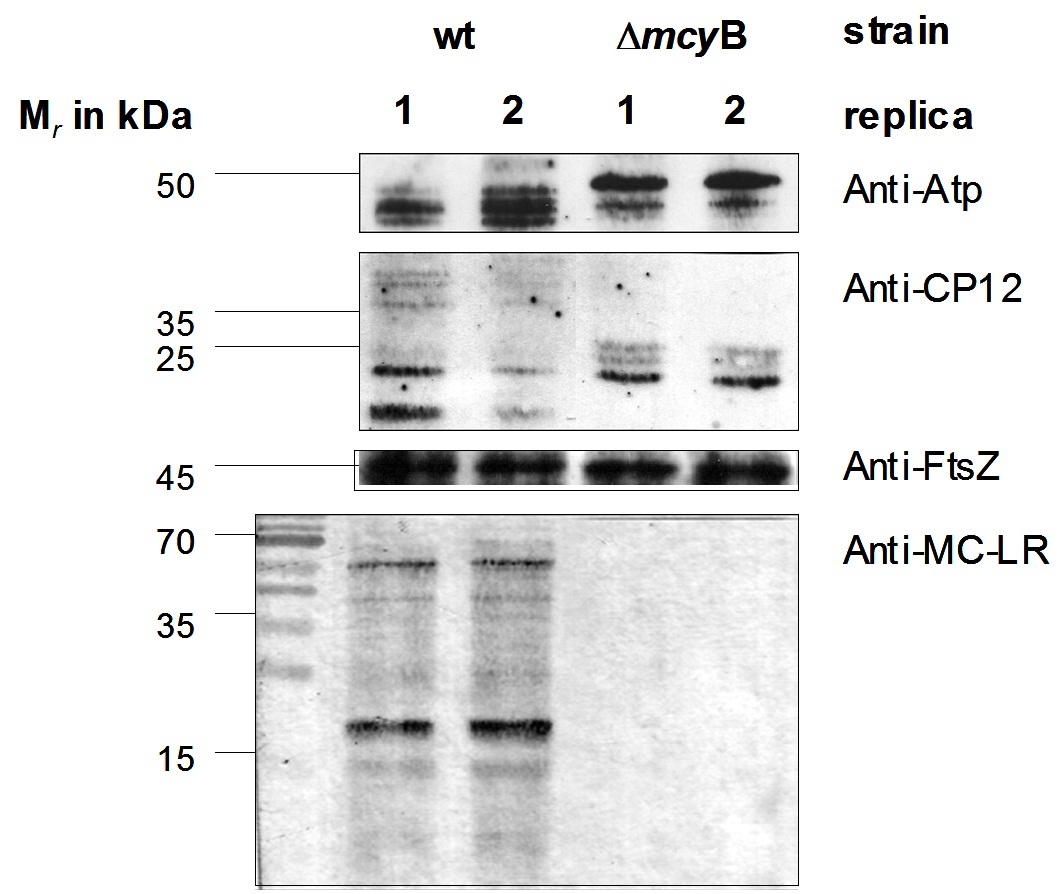

Supplement: Figure S1 — Immunoblot analysis of soluble fraction of Microcystis aeruginosa PCC 7806 wildtype (wt) and microcystin-deficient mutant ΔmcyB after exposition to dark condition for 2 hours. Blots were obtained after separation (15% SDS-PAGE) and transfer of soluble protein extracts (40 µg) to nitrocellulose membranes. Equality of the samples was controlled by Ponceau staining of the membrane as well as Coomassie staining of the duplicated gel. Immunoblot analysis was performed for selected differential protein spots with antibodies against ATP synthase, CP12 like polypeptide, FtsZ (used as loading control) and microcystin, respectively. (JPG) [file pone.0017615.s002.jpg]

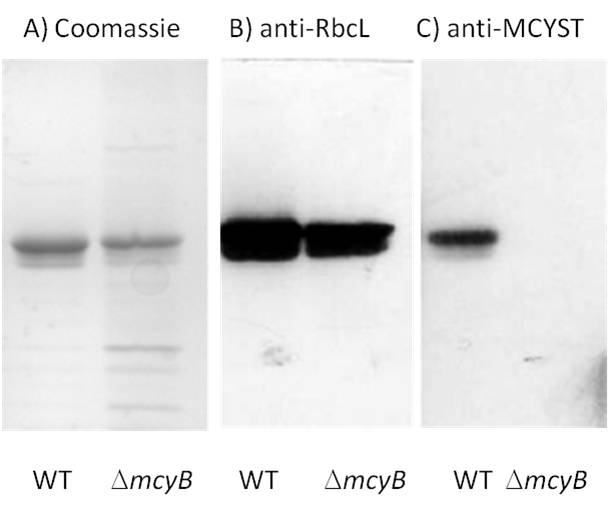

Supplement: Figure S2 — Microcystin-binding in carboxysome-enriched fraction that was obtained by fractionated solubilization. A) Coomassie stained fractions of wild type and ΔmcyB mutant. B) Immunoblot analysis using anti-RbcL antibody (identity of RbcL was further confirmed using mass spectrometry). C) Immunoblot analysis using anti-microcystin antibody. (JPG) [file pone.0017615.s003.jpg]

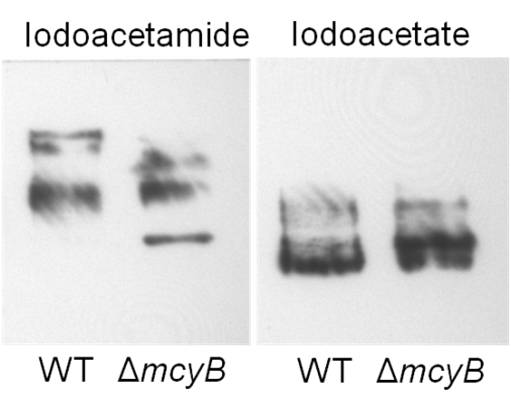

Supplement: Figure S3 — Differential alkylation pattern of RbcL of PCC 7806 wild type and ΔmcyB mutant, respectively, with iodoacetamide and iodoacetate leading to charge differences depending on the presence of free thiol groups. RbcL fractions were run on native PAGE and subsequently evaluated by immunoblot analysis with an RbcL specific antibody. (JPG) [file pone.0017615.s004.jpg]

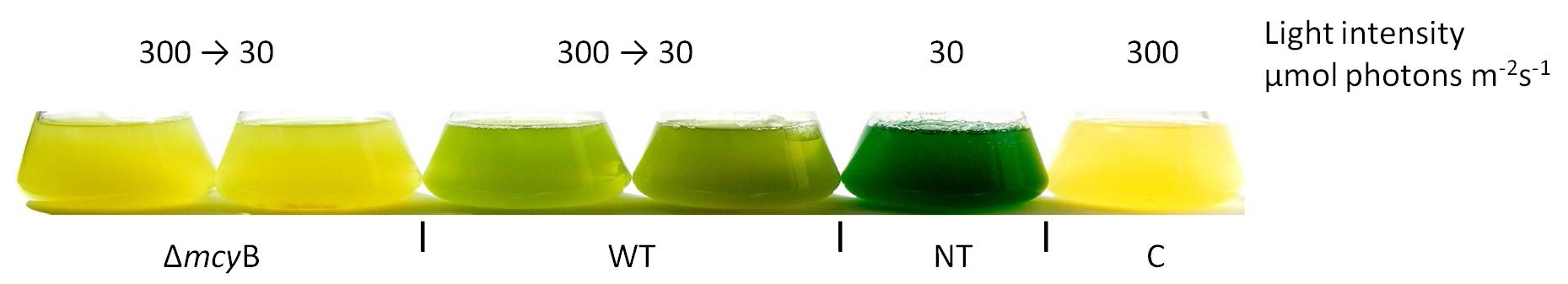

Supplement: Figure S4 — Recovery of ΔmcyB mutant and wild type after one week irradiation with 300 µmol photons m−2 s−1 and subsequent transfer to 30 µmol photons m−2 s−1 for three days. NT, non-treated control of wild type; C, control treated for one week with 300 µmol photons m−2 s−1 without recovery of wild type. (JPG) [file pone.0017615.s005.jpg]
